# Cooccurrence of NDM-1, ESBL, RmtC, AAC(6′)-Ib, and QnrB in Clonally Related* Klebsiella pneumoniae* Isolates Together with Coexistence of CMY-4 and AAC(6′)-Ib in* Enterobacter cloacae* Isolates from Saudi Arabia

**DOI:** 10.1155/2019/6736897

**Published:** 2019-07-29

**Authors:** Mohamed H. Al-Agamy, Taghrid S. El-Mahdy, Hesham H. Radwan, Laurent Poirel

**Affiliations:** ^1^Department of Pharmaceutics, College of Pharmacy, King Saud University, 11451 Riyadh 2457, Saudi Arabia; ^2^Microbiology and Immunology Department, Faculty of Pharmacy, Al-Azhar University, Nasr City, Cairo, Egypt; ^3^Department of Microbiology and Immunology, Faculty of Pharmacy, Helwan University, Cairo, Egypt; ^4^Department of Microbiology, Faculty of Pharmacy, Ahram Canadian University, Cairo, Egypt; ^5^Medical and Molecular Microbiology Unit, Section of Medicine, Faculty of Science and Medicine, University of Fribourg, Switzerland

## Abstract

The aim of this study was to investigate the mechanisms responsible for resistance to antimicrobials in a collection of enterobacterial isolates recovered from two hospitals in Saudi Arabia. A total of six strains isolated from different patients showing high resistance to carbapenems was recovered in 2015 from two different hospitals, with four being* Klebsiella pneumoniae* and two* Enterobacter cloacae. *All isolates except one* K. pneumoniae* were resistant to tigecycline, but only one* K. pneumoniae *was resistant to colistin. All produced a carbapenemase according to the Carba NP test, and all were positive for the EDTA-disk synergy test for detection of MBL. Using PCR followed by sequencing, the four* K. pneumoniae* isolates produced the carbapenemase NDM-1, while the two* E. cloacae* isolates produced the carbapenemase VIM-1. Genotyping analysis by Multilocus Sequence Typing (MLST) showed that three out of the four* K. pneumoniae *isolates were clonally related. They had been recovered from the same hospital and belonged to Sequence Type (ST) ST152. In contrast, the fourth* K. pneumoniae* isolate belonged to ST572. Noticeably, the NDM-1-producing* K. pneumoniae* additionally produced an extended-spectrum ß-lactamase (ESBL) of the CTX-M type, together with OXA-1 and TEM-1. Surprisingly, the three clonally related isolates produced different CTX-M variants, namely, CTX-M-3, CTX-M-57, and CTX-M-82, and coproduced QnrB, which confers quinolone resistance, and the 16S rRNA methylase RmtC, which confers high resistance to all aminoglycosides. The AAC(6′)-Ib acetyltransferase was detected in both* K. pneumoniae* and* E. cloacae*. Mating-out assays using* Escherichia coli *as recipient were successful for all isolates. The *bla*_NDM-1_ gene was always identified on a 70-kb plasmid, whereas the *bla*_VIM-1_ gene was located on either a 60-kb or a 150-kb plasmid the two* E. cloacae* isolates, respectively. To the best of our knowledge, this is the first report of the coexistence of an MBL (NDM-1), an ESBL (CTX-M), a 16S rRNA methylase (RmtC), an acetyltransferase (AAC[6′]-Ib), and a quinolone resistance enzyme (QnrB) in* K. pneumoniae *isolates recovered from different patients during an outbreak in a Saudi Arabian hospital.

## 1. Introduction

Metallo-*β*-lactamases (MBLs) are enzymes that hydrolyze most *β*-lactams including carbapenems, the most potent *β*-lactams. Therefore, resistance mediated by MBLs is considered the most serious mechanism for inactivating *β*-lactams [1]. Of the clinically relevant MBLs, NDM, VIM, and IMP are the most common worldwide [2, 3].* Klebsiella pneumoniae *and* Enterobacter cloacae *are* Enterobacteriaceae* that are leading causes of nosocomial infections and can frequently acquire antibiotic resistance [4].* K. pneumoniae *and* E. cloacae* can acquire resistance to third-generation cephalosporins through plasmid-mediated AmpC *β*-lactamases, such as CMY, FOX, ACC, LAT, MIR, MOX, DHA, and ACT [5], as well as extended-spectrum *β*-lactamases (ESBL), such as CTX-M, TEM-, and SHV-derivatives. Acquisition of carbapenem resistance in those bacterial species may be related to several carbapenem-hydrolyzing ß-lactamases, such as KPC, NDM, VIM, and OXA-48 [6]. Several mechanisms may confer aminoglycoside resistance to* Enterobacteriaceae*, including production of aminoglycoside-modifying enzymes, such as aminoglycoside acetyltransferases (AACs), phosphotransferases (APHs), and adenylyltransferases (ANTs), but also target modifications by mutations in ribosomal proteins, or 16S rRNA methylation through the action of 16S rRNA methylases [7]. AACs are the most common aminoglycoside-modifying enzymes and are subdivided into four groups [AAC(1), AAC(2′), AAC(3), and AAC(6′)]. AAC(6′) comprises the most common enzymes in Gram-negative bacteria and can be subdivided into two groups: AAC(6′)-I and AAC(6′)-II [8, 9].* Enterobacteriaceae* may also produce 16S rRNA methylases, including ArmA, RmtA to RmtH, and NpmA, which confer resistance to aminoglycosides [10]. The plasmid-mediated quinolone resistance genes* qnrA, qnrB, qnrC, qnrD, qnrS, *and* qnrVC *code for Qnr proteins that protect DNA gyrase and topoisomerase IV from the action of quinolones, conferring quinolone resistance [11]. In this study, we aimed to investigate the mechanisms responsible for carbapenem resistance in a collection of enterobacterial isolates recovered from two hospitals in Saudi Arabia. Carbapenemase-producing isolates were also further investigated for additional antibiotic resistance traits.

## 2. Materials and Methods

### 2.1. Bacterial Isolates

Four* K. pneumoniae* and two* E. cloacae* isolates from two hospitals in Riyadh, the capital of Saudi Arabia and Al-Gouf, the north region of Saudi Arabia were highly resistant to carbapenems. Three* K. pneumoniae* isolates (KP-Q1, KP-Q2, and KP-Q3) were obtained from hospital A while a single* K. pneumoniae* (KP-Q4) and two* E. cloacae* (EN.C.Q5 and EN.C.Q6) isolates were collected from hospital B in 2015 (Table 1). Isolates were identified in the clinical laboratory using the VITEK 2 system (bioMérieux, Marcy l'Etoile, France) and confirmed by conventional morphological and biochemical tests.

### 2.2. Antimicrobial Susceptibility Testing and Phenotypic Carbapenemase Detection

Etest strips (bioMérieux,Marcy l'Etoile, France) and disk diffusion methods (Oxoid, UK) were used to determine susceptibility to antimicrobials other than colistin, whose susceptibility was tested using a microbroth dilution method. The guidelines of the Clinical Laboratory Standards Institute (CLSI, 2016) [12] were used to interpret MICs except for tigecycline and colistin, whose data were interpreted according to the 2019 guidelines of the European Committee on Antimicrobial Susceptibility Testing (EUCAST) [13]. The Carba NP test [14] was carried out to detect carbapenemase activity. Production of MBL was screened by an EDTA-disk synergy test [15].

### 2.3. PCR Amplification of Resistance Genes

Carbapenem-resistant enterobacterial isolates were collected from 200 *μ*L volumes of overnight Luria Bertani broth (Thermo Fischer Scientific, Waltham, MA, USA) cultures and resuspended in equal volumes of sterile distilled water, heated at 99°C for 10 min on a heating block, and then centrifuged at 15,000 rpm for two minutes. The supernatant, which contained released DNA, was used directly in PCR amplification of the resistance genes on a Techne Flexigene Thermocycler (Techne, Duxford, Cambridge, UK). Positive and negative controls were included in all PCR runs. All PCR amplicons were electrophoresed on 0.8% agarose gels containing 0.5 mg/L ethidium bromide and then analyzed under UV light (Pharmacia LKB; Biotechnology AB, Gothenburg, Sweden) and photographed using a documentation system.

The multiplex PCR protocol described by Dallenne et al. [16] was used to amplify four types of class A *β*-lactamase genes (TEM, SHV, five CTX-M families, and an OXA-1-like). Multiplex PCR was used to seek plasmid-mediated AmpC *β*-lactamase genes, including ACC, FOX, MOX, CMY, LAT, and MIR, as previously described [17] using six pairs of primers. Ten carbapenemase genes, including IMP, VIM, NDM, SIM, SPM, GIM, AIM, DIM, OXA-48, and KPC, were tested via three multiplex reactions according to the method of Poirel et al. [18]. Moreover, the isolates were screened by multiplex PCR for* qnrA*,* qnrB*, and* qnrS* plasmid-mediated quinolone resistance genes [19], whereas a simplex PCR was performed to detect AAC(6′)-Ib [7]. Additionally, six 16S rRNA methylase genes were tested using two multiplex reactions, with the first detecting* armA*,* rmtB*, and* npmA* and the second* rmtA*,* rmtC*, and* rmtD* according to the previously published method [20]. The PCR products were sequenced and analyzed using the NCBI database with the BLAST program (http://www.ncbi.nlm.nih.gov).

### 2.4. Multilocus Sequence Typing (MLST) Analysis

Clonal relatedness was determined by MLST according to the Pasteur Institute scheme for the* K. pneumoniae* isolates (https://bigsdb.pasteur.fr/klebsiella/klebsiella.html) and according to the PubMLST protocol and database for the* E. cloacae* isolates (https://pubmlst.org/ecloacae/).

### 2.5. Plasmid Extraction and Conjugation Experiment

A plasmid extraction protocol [21] was used to investigate the presence of plasmids in all isolates and was followed by direct agarose gel electrophoresis of extracted DNA. Bac-tracker (Epicentre, Madison, WI, USA) was used as a plasmid size marker.

Transfer of imipenem resistance genes by conjugation from our isolates as donors was attempted by filter mating [22] with* Escherichia coli* J53 sodium azide resistant as the recipient strain. The initial donor/recipient ratio was 1:5 or 1:10. Transconjugants were selected on MacConkey agar containing sodium azide (100 mg/L) and ceftazidime (4 mg/L).

## 3. Results

MICs of different antibiotics for the six carbapenem-resistant* K. pneumoniae* and* E. cloacae* isolates are shown in Table 2. All isolates were highly resistant to all tested *β*-lactams. They all remained susceptible to tigecycline (MIC ≤ 2 mg/L) according to the EUCAST 2016 breakpoint, although the cut-off tigecycline in EUCAST 2019 guidelines is 0.5 mg/L. Consequently, all our isolates, except one* K. pneumonia*, were resistant to tigecycline using the new EUCAST definition. A single* K. pneumoniae* isolate (KP-Q2) was resistant to colistin according to the EUCAST definition (MIC > 2 mg/L is considered resistant). Moreover, the four* K. pneumonia*e isolates were determined to be susceptible to chloramphenicol with inhibition zone diameters > 12 mm according to the 2016 CLSI guidelines, whereas the two* E. cloacae* isolates were resistant to this compound. Resistance to sulfamethoxazole/trimethoprim was also observed in all isolates. Four isolates (three* K. pneumoniae* and a single* E. cloacae*) were resistant to tetracycline. Resistance profiles of aminoglycosides (amikacin and gentamicin) and quinolones/fluoroquinolones (nalidixic acid, ciprofloxacin, and ofloxacin) are presented in Table 2.

All isolates exhibited a carbapenemase activity as detected by the Carba NP test. MBL production was detected by EDTA-disk synergy tests in all isolates. This was confirmed by PCR, with the *bla*_NDM-1_ carbapenemase gene being amplified in the four* K. pneumoniae *isolates and the *bla*_VIM-1_ gene being amplified in the two* E. cloacae* isolates (Table 3). No additional carbapenemase gene was identified in those six isolates.

Additionally, three* K. pneumoniae *isolates harbored a 16S rRNA methylase encoding gene,* rmtC*, and the quinolone resistance gene,* qnrB*. Moreover, a single* E. cloacae *isolate harbored the plasmid-mediated AmpC-encoding genes, *bla*_CMY-4_. Additionally, all isolates were found to harbor CTX-M-ESBL-encoding genes. All* K. pneumoniae* isolates were found to harbor OXA-1 broad spectrum beta-lactamases (BSBL). TEM-1-BSBL was found in all isolates except* E. cloacae* isolate EN.C.Q6. The aminoglycoside-modifying enzyme AAC(6′)-Ib was found in all isolates except* E. cloacae* isolate EN.C.Q5 (Table 3).

**Figure 1:**
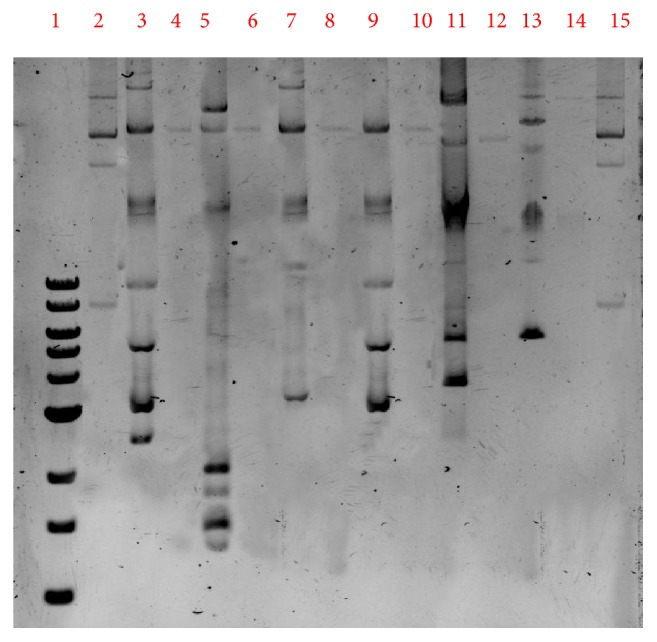
Electrophoretic profile of the plasmid DNA extracts from the strains and their transconjugants to confirm the results. 1. Ladder 1kb (10kb,8kb,6kb,5kb,4kb,3kb,2kb,1.5kb,1kb). 2. Ladder control strain 50192 (154kb, 66kb, 48kb, 7.8kb). 3. KP1 strain (70kb). 4. KP1 Transconjugant strains (70kb). 5. KP 2 (70kb). 6. KP2 Transconjugant strains (70kb). 7. KP 3 (70kb). 8. KP3 Transconjugant strains (70 kb). 9. KP 4 strains (70kb). 10. KP 4 Transconjugant strain 4 (70 kb). 11. EN.C 5 strains (60 kb). 12. EN.C 5 Transconjugant strain (60kb). 13. EN.C 6 strain (150kb). 14. EN.C6 Transconjugant strains (150kb). 15. Ladder control strain 50192 (154kb, 66kb, 48kb, 7.8kb).

Transfer of imipenem resistance from all isolates to* E. coli* J53 was attempted by filter mating. The conjugation experiment demonstrated conjugative transfer of *bla*_NDM-1_ from* K. pneumoniae* and *bla*_VIM-1_ from* E. cloacae* to* E. coli* J53, confirming carriage of both genes on transferable plasmids. In* K. pneumoniae*, a single 70-kb plasmid was identified in all four isolates, onto which the *bla*_NDM-1_ gene was located. On the other hand, the *bla*_VIM-1_ gene was located on a 60-kb plasmid in a single* E. *cloacae isolate and on a 150-kb plasmid in the other isolate (Figure 1).

MLST genotyping revealed that three* K. pneumoniae* isolates, which had actually been recovered from same hospital, belonged to ST152. The fourth* K. pneumoniae* isolate belonged to ST572 and had been recovered from the other hospital.

## 4. Discussion

The overuse of carbapenems during the last decade has led to increasing levels of bacterial resistance toward these potent *β*-lactams. Enterobacterial isolates have shown high carbapenem resistance rates worldwide, making alternative antibiotics, such as colistin and tigecycline, urgently needed. In the current study, we investigated by using phenotypic and genotypic methods six carbapenem-resistant enterobacterial isolates for their multiresistance determinants. In a recent study from Saudi Arabia [23], tigecycline resistance was not detected among 31* Enterobacteriaceae* clinical isolates (21* K. pneumoniae* and 10* E. coli*) whereas colistin resistance was seen in 10% (one isolate) and 4.8% (one isolate) of their* E. coli* and* K. pneumonia* isolates, respectively. Similarly, colistin and tigecycline susceptibility were 100 and 87.5%, respectively, in 16 extensively drug-resistant* K. pneumoniae* strains from Saudi Arabia [24]. According to several reports [25–27], colistin and tigecycline remain the most effective antibiotics against carbapenem-resistant Gram-negative pathogens. Interestingly, the 2019 EUCAST guidelines (http://www.eucast.org/fileadmin/src/media/PDFs/EUCAST_files/Breakpoint_tables/v_9.0_Breakpoint_Tables.pdf) [13] changed the tigecycline resistance breakpoint to MIC > 0.5 mg/L. According to this new definition, all our isolates except one* K. pneumoniae* isolate would be considered resistant, suggesting an increasing challenge to public health. In the current study, resistance to colistin is not determined in our isolates, except one* K. pneumonia* isolate which has low-level colistin resistance (MIC 3 mg/L).

Two members of MBLs were detected among our isolates, namely, NDM-1-producing* K. pneumoniae* isolates and VIM-1-producing* E. cloacae* isolates. Although OXA-48 is commonly identified among* K. pneumoniae* isolates in some parts of Saudi Arabia [28–30], this resistance determinant was not detected in the present study. Moreover, a multicenter study in Saudi Arabia, Memish et al. [28], reported that OXA-48 and NDM-1 are the dominant carbapenemases among 124* Enterobacteriaceae* (*E. coli*,* Klebsiella* spp., and* Enterobacter* spp.) isolated from 12 cities across the Kingdom of Saudi Arabia with low incidence of VIM and complete absence of KPC and IMP.

The coexistence of 16S rRNA methylase genes with *β*-lactamase genes was previously observed in Saudi Arabia. ESBLs were reported along with* armA, rmtB, rmtC, *and* npmA *in* Enterobacteriaceae* [31]. OXA-48 and NDM were detected along with* armA* and* rmtB* in* K. pneumoniae* [24]. The current study also revealed that the three clonally related* K. pneumoniae* isolates harbored the* rmtC* gene along with *bla*_NDM-1_. In addition, the plasmid mediated AmpC *β*-lactamase variant, CMY-4, was determined in our study in one* E. cloacae *which coproduces VIM-1 as well. This finding is the first report describing CMY carriage by a clinical* E. cloacae* isolate from Saudi Arabia.

The gene encoding the aminoglycoside-modifying enzyme AAC(6′)-Ib was detected in all but one isolates in our study. This enzyme was previously detected among extensively drug-resistant* K. pneumonia* isolates in Saudi Arabia [24]. Furthermore, the gene encoding the quinolone resistance protein QnrB was detected in the* K. pneumoniae* isolates from our study, in accordance with previous reports from Saudi Arabia [23, 24].

Noteworthy, strain EN.C.Q5 of our* E. cloacae* isolates was resistant to gentamicin although it did not have any of aminoglycoside resistance determinant examined (16S rRNA methylase:* armA*,* rmtA*,* rmtB*,* rmtC*,* rmtD*, and* npmA, or acetyltransferase* AAC(6′)-Ib), suggesting other resistance mechanism involved.* Similarly*,* K. pneumonia strain* KP-Q4 and* E. cloacae* strain EN.C.Q6 were resistant to ciprofloxacin and ofloxacin without* qnrA*,* qnrB*, and* qnrS* plasmid mediated quinolone resistance genes. Quinolone resistance in these isolates may be due to other plasmid mediated genes or mutations in quinolone resistance–determining regions of DNA gyrase (*gyrA* and* gyrB*) or DNA topoisomerase IV (*parC* and* parE*) [32].

## 5. Conclusions

Our study reports on a series of threatening resistance determinants responsible for the multidrug resistance pattern observed among clinical isolates. The identification of MBL-encoding genes (*bla*_VIM_ in* E. cloacae* and *bla*_NDM_ in* K. pneumoniae*) being identified onto conjugative plasmids raises concerns about the real extend of diffusion of those resistance genes in Saudi Arabia. Cooccurrence of multiple resistance determinants in clonally related* K. pneumoniae* isolates highlights the importance of controlling the dissemination of such isolates by early detection in hospital settings.

## Figures and Tables

**Table 1:** Isolate case histories and characteristics.

Isolate	Hospital/City	Patient age (years)	Patient sex	Specimen source	Diagnosis
KP-Q1	A/ Al-Gouf	65	Male	Blood	Septic encephalopathy
KP-Q2	A/ Al-Gouf	95	Male	Sputum	Pulmonary fibrosis
KP-Q3	A/ Al-Gouf	70	Female	Wound	Gangrene of diabetic foot with sepsis
KP-Q4	B/ Riyadh	50	Male	Wound	Urogenital infection
EN.C.Q5	B/ Riyadh	64	Female	Blood	ND
EN.C.Q6	B/ Riyadh	78	Male	Blood	Cystic fibrosis

KP: *Klebsiella pneumoniae*

EN.C: *Enterobacter cloacae*

ND: Not determined

**Table 2:** Antimicrobial resistance profile of six resistant *Klebsiella pneumoniae *and *Enterobacter cloacae *isolates.

Antibiotics	MIC (mg/L)					
	KP-Q1	KP-Q2	KP-Q3	KP-Q4	EN.C.Q5	EN.C.Q6
Amoxicillin	>256	>256	>256	>256	>256	>256
Piperacillin	>256	>256	>256	>256	>256	>256
Ticarcillin	>256	>256	>256	>256	>256	>256
Cefoperazone	>256	>256	>256	>256	>256	>256
Cefotaxime	>256	>256	>256	>256	>256	>256
Ceftazidime	>256	>256	>256	>256	>256	>256
Cefepime	>256	>256	>256	>256	>256	>256
Aztreonam	>256	>256	>256	>256	>256	>256
Cefoxitin	>256	>256	>256	>256	>256	>256
Cefotetan	>256	>256	>256	>256	>256	>256
Imipenem	>256	>256	>256	>256	>256	>256
Meropenem	>32	>32	>32	>32	>32	>32
Doripenem	>32	>32	>32	>32	>32	>32
Amikacin	>256	>256	>256	>256	24 (S)	>256
Gentamicin	>256	>256	>256	>256	64	>256
Nalidixic acid	>256	>256	>256	>256	>256	>256
Ciprofloxacin	>32	>32	>32	>32	2 (S)	8
Ofloxacin	>32	>32	>32	>32	2 (S)	8
Tigecycline	0.75	0.75	0.75	0.19 (S)	2	2
Colistin	0.75 (S)	3	1.5 (S)	2 (S)	0.75 (S)	0.75 (S)
	Inhibition zone diameter (mm) by disk diffusion					
Sulfamethoxazole/trimethoprim 23.75 / 1.25 *µ*g	6	6	6	6	6	6
Tetracycline 30 *μ*g	6	6	8	15 (S)	6	12 (I)
Chloramphenicol 30 *μ*g	22 (S)	23 (S)	21 (S)	16 (S)	6	6

KP: *Klebsiella pneumoniae*, EN.C: *Enterobacter cloacae*

S: susceptible, I: intermediate

Disk diameter is 6 mm

MIC: Minimum Inhibitory Concentration

Resistance interpretation for all antimicrobials unless labeled S or I

Interpretation according to CLSI guidelines (2016) except for tigecycline and colistin, which were interpreted according to EUCAST guidelines (2019).

**Table 3:** Antibiotic resistance enzymes and genotypic screening of six resistant *Klebsiella pneumonia *and *Enterobacter cloacae *isolates.

Strain	Carbapenemase	Size of plasmids harboring carbapenemase-encoding genes	ESBL	BSBL	16S rRNA methylase	AmpC variant	Aminoglycoside-modifying enzyme (acetyltransferase)	Quinolone resistance enzymes	MLST
KP-Q1	NDM-1	70 kb	CTX-M-3	TEM-1, OXA-1	RmtC	-	AAC(6′)-Ib	QnrB	ST152
KP-Q2	NDM-1	70 kb	CTX-M-57	TEM-1, OXA-1	RmtC	-	AAC(6′)-Ib	QnrB	ST152
KP-Q3	NDM-1	70 kb	CTX-M-82	TEM-1, OXA-1	RmtC	-	AAC(6′)-Ib	QnrB	ST152
KP-Q4	NDM-1	70 kb	CTX-M-15	TEM-1, OXA-1	-	-	AAC(6′)-Ib	-	ST572
EN.C.Q5	VIM-1	60 kb	-	TEM-1	-	CMY-4	-	-	ST171
EN.C.Q6	VIM-1	150 kb	-	-	-	-	AAC(6′)-Ib	-	ST73

KP: *Klebsiella pneumoniae*

EN.C: *Enterobacter cloacae*

ESBL: Extended-spectrum *β*-lactamase

MLST: Multilocus sequence typing

## Data Availability

The data used to support the findings of this study are available from the corresponding author upon request.
